# HPV Prevalence and Prognostic Value in a Prospective Cohort of 255 Patients with Locally Advanced HNSCC: A Single-Centre Experience

**DOI:** 10.1155/2013/437815

**Published:** 2013-04-24

**Authors:** E. Thibaudeau, B. Fortin, F. Coutlée, P. Nguyen-Tan, X. Weng, M.-L. Audet, O. Abboud, L. Guertin, A. Christopoulos, J. Tabet, D. Soulières

**Affiliations:** ^1^Department of Head and Neck Surgery, Centre Hospitalier de l'Université de Montréal, Hôpital Notre-Dame, 1560 Sherbrooke Est, Montreal (Quebec), Canada H2L 4M1; ^2^Department of Radiation Oncology, Hôpital Maisonneuve-Rosemont, 5415 Boulevard de l'Assomption, Montréal (Quebec), Canada H1T 2M4; ^3^Department of Microbiology, Centre Hospitalier de l'Université de Montréal, Hôpital Notre-Dame, 1560 Sherbrooke Est, Montreal (Quebec), Canada H2L 4M1; ^4^Department of Radiation Oncology, Centre Hospitalier de l'Université de Montréal, Hôpital Notre-Dame, 1560 Sherbrooke Est, Montreal (Quebec), Canada H2L 4M1; ^5^Department of Haematology and Medical Oncology, Centre Hospitalier de l'Université de Montréal, Hôpital Notre-Dame, 1560 Sherbrooke Est, Montreal (Quebec), Canada H2L 4M1; ^6^Laboratoire de Biologie Moléculaire et Hématologie Spéciale, Département d'Hématologie, Hématologue et Oncologue Médical, Centre Hospitalier de l'Université de Montréal, Hôpital Notre-Dame, 1560 Sherbrooke Est, Montreal (Quebec), Canada H2L 4M1

## Abstract

*Background*. HPV is a positive prognostic factor in HNSCC. We studied the prevalence and prognostic impact of HPV on survival parameters and treatment toxicity in patients with locally advanced HNSCC treated with concomitant chemoradiation therapy. *Methods*. Data on efficacy and toxicity were available for 560 patients. HPV was detected by PCR. Analysis was performed using Kaplan-Meier survival curves, Fisher's test for categorical data, and log-rank statistics for failure times. *Results*. Median follow-up was 4.7 years. DNA extraction was successful in 255 cases. HPV prevalence was 68.6%, and 53.3% for HPV 16. For HPV+ and HPV−, median LRC was 8.9 and 2.2 years (*P* = 0.0002), median DFS was 8.9 and 2.1 years (*P* = 0.0014), and median OS was 8.9 and 3.1 years (*P* = 0.0002). Survival was different based on HPV genotype, stage, treatment period, and chemotherapy regimen. COX adjusted analysis for T, N, age, and treatment remained significant (*P* = 0.004). *Conclusions*. Oropharyngeal cancer is increasingly linked to HPV. This study confirms that HPV status is associated with improved prognosis among H&N cancer patients receiving CRT and should be a stratification factor for clinical trials including H&N cases. Toxicity of CRT is not modified for the HPV population.

## 1. Introduction

Tobacco and alcohol consumption has long been known as the major risk factor for HNSCC. However, HPV has recently been recognised to play a role in the pathogenesis of a subset of clinically and molecularly distinct HNSCC, most often located in the oropharynx and associated with wild-type p53 and downregulation of cyclin D and retinoblastoma protein pRb [1–5], and in which viral oncoproteins E6 and E7 play a crucial part [6]. 

 HPV prevalence in HNSCC has been increasing significantly in the past few decades [5, 7]; it is estimated at 25% in HNSCC [8], but reaches up to 70% or more in tonsillar SCCs [9–11]. Unlike the HPV-negative oropharyngeal cancers, the HPV-positive subset is not associated with tobacco or alcohol use, but with certain types of sexual behaviours [12, 13]. The HPV 16 subtype is present in up to 90% of HPV-related oropharyngeal cancers, while HPVs 18, 31, and 33 have been identified in the remainder [14, 15]. HPV has recently been recognised as a good prognostic factor in head and neck (H&N) cancer [5, 16–26], which has been attributed to several mechanisms, including absence of field cancerisation and increased sensitivity to chemoradiation therapy [5, 16, 20, 22–24, 26]. 

Most of the available data is derived from small randomised trials with different treatment options or small heterogeneous cohorts; moreover, data were often collected retrospectively. Even though one prospective clinical trial concluded with the same prognostic advantage [16], other favourable prognostic factors associated with HPV positivity, such as younger age or early tumour stages, could not be ruled out entirely. The study presented here evaluates the prevalence and prognostic impact of HPV on overall survival (OS), disease-free survival (DFS), local-regional control (LRC), and treatment toxicity, in a large cohort of consecutive patients with locally advanced HNSCC, treated with concomitant platinum-based chemoradiation therapy (CRT) and followed prospectively. A more specific focus is placed on oropharyngeal squamous cell carcinoma, as previous literature has demonstrated that the prognostic impact of HPV is most important in this subsite, which also represents the largest group in our HNSCC population.

## 2. Materials and Methods

### 2.1. Study Design and Eligibility

The present series comprises cases from patients participating in an ongoing tumor bank of patients treated for HNSCC at CHUM Hôpital-Notre-Dame since 1998. Eligibility criteria included locally advanced HNSCC and treatment with primary chemoradiation and with a minimal followup of three years. Surgical treatment preceding chemoradiation was the main exclusion criterion. Data were collected prospectively from a regular assessment of outcome variables such as response rates, local or regional recurrences, and survival rates by means of regular clinical and radiological evaluations. All patients had histological confirmation of SCC based on histological features in hematoxylin and eosin-stained tissue sections diagnosed by a pathologist experienced in head and neck pathology. Staging was performed according to the TNM classification system from clinical and radiological assessment.

### 2.2. Patient Population

All patients with locally advanced HNSCC stage III-IVA-IVB treated with radical radiation therapy (min 7000cGY standard fractionation or altered fractionation) and concurrent chemotherapy (Cisplatin 100 mg/m^2^ q 3 weeks × 3 or Carboplatin 70 mg/m^2^ d1-4 + 5-FU 600 mg/m^2^ d1-4 q 3 weeks × 3 or Cisplatin 6 mg/m^2^ daily or Carboplatin 25 mg/m^2^ daily) were included in this analysis. Patients were secondarily selected based on the availability of tumor samples (cf.  Table 1).

### 2.3. Sample Preparation

Three to eight sections of 10 *μ*m were obtained from each tumor. To avoid cross-contamination during sectioning, disposable microtome blades were used, and the microtome was cleaned after cutting each specimen. Biopsy specimens were fixed in 10% formalin solution and processed according to conventional methods for paraffin-embedded histological sections for routine diagnosis. 

### 2.4. Overview

Prior to HPV detection, samples underwent PCR for detection of *β*-globin using the PCO4/GH2O method (268-base pair primers) to control for DNA integrity and for the absence of competing inhibitors. 

Samples were then tested for the presence of HPV DNA using the Roche Linear Array detection method (LA-HPV) (primers 450 bp).

Samples that tested negative for HPV-DNA with the LA-HPV technique were tested using the GP5+/GP6+ PCR detection method (primers 150 bp).

Samples that tested negative for both HPV-DNA and *β*-globin with the LA-HPV detection method, and also negative for HPV DNA using the GP5+/GP6+ technique, were also tested for the presence of *β*-globin using PCO3/PCO4 probes (110 bp) to ensure that negative results were not caused by excessive DNA fragmentation due to extended preservation in paraffin.

### 2.5. DNA Extraction

Paraffin-embedded samples were heated to 72°C and washed with xylene for two minutes, four times. The samples were then submitted to four one-minute washes with ethanol 100% and four one-minute washes with ethanol 95%. Remaining tissue was then incubated in 200 *μ*L lysis buffer (10 mM tris-HCl, pH 8.0, 1 mM EDTA, pH 8.0, and 20 mM NaCl) containing 0.2 mg/mL proteinase K for 2 hours at 55°C. The mixture was then heated at 96°C for 5 minutes in order to inactivate proteinase K. Optic density was calculated for the supernate after having centrifuged the mixture at 12000 G for 20 minutes. Tubes were then stored at −4°C.

### 2.6. PCO4/GH2O PCR

Amplification of a *β*-globin gene fragment was performed by the use of PCO4 and GH2O primers to control for target DNA integrity.

PCR products were separated by electrophoresis on a 2% ethidium-stained agarose gel and visualised on a UV transilluminator. Samples generating a visible 268-base pair band were judged suitable for detection of HPV DNA using the LA-HPV method [27], which includes detection of *β*-globin using the PCO4/GH2O method.

### 2.7. LA-HPV

As previously described [28] PCR was performed in a final reaction volume of 100 *μ*L with 5 *μ*L of sample material and 95 *μ*L of kit working master mix (containing MgCl_2_, KCl, Ampli*Taq*, gold DNA polymerase (Ampli*Taq*; Perkin-Elmer; Foster City, CA), uracil-*N*-glycosylase, dATP, dCTP, dGTP, dUTP, dTTP, and biotinylated PGMY primers and *β*-globin primers GH2O and PCO4). Test tubes were incubated in a TC 9700 thermal cycler set at maximum ramp speed for 2 minutes at 50°C and 9 minutes at 95°C, followed by 40 cycles of 30 seconds at 95°C, 1 minute at 55°C, and 1 minute at 72°C, with a final extension at 72°C (ramp set at 50%) for 5 minutes.

Negative and positive controls were included for each reaction.

Amplicons were denatured in 0.4 N NaOH and hybridise to an immobilised probe array containing probes for 37 HPV genotypes (according to the protocol provided by Roche Molecular Systems). 

Following the hybridization reaction, Linear Array HPV Genotyping Strips were stringently washed to remove unbound material, and positive hybridization reactions were detected by streptavidin-horseradish peroxidase-mediated color precipitation on the membrane at the probe line.

The probe for detection of HPV 52 amplicons was a cross-reactive probe that also hybridised with types 33, 35, and 58; samples positive with the HPV 52 probe and containing at least one of those types were thus also tested with a real-time PCR assay specific for HPV 52 (see below). Only samples positive for HPV 52 with real-time PCR were considered HPV 52 positive.

### 2.8. Real-Time PCR Assay for HPV 52

As described previously [28], 20 *μ*L reaction mixtures contained 10 mM tris-HCl; pH 8.0; 50 mM KCl; a 200 *μ*M concentration of (each) ATP, dGTP, and dCTP; 400 *μ*M dUTP; 0.05 *μ*M of TaqMan probe 52-TM (CGTGCAGGGTCCGGGGTC); 0.3 pmol each of primers 52JA-3 (GAACACAGTGTAGCTAACGCACG) and 52JA-4 (GCATGACGTTACACTTGGGTCA) (targeting the E6 gene); 2.0 mM MgCl_2_; and 5 units of Ampli*Taq* gold DNA polymerase. Capillaries were placed in a LightCycler system (Roche Molecular Systems; Branchburg, NJ, USA) and amplified at 95°C for 10 minutes, followed by 50 cycles at 95°C for 15 seconds and 60°C for 60 seconds. Ten copies of an HPV 52-expressing plasmid in 500 ng of cellular DNA served as a weak positive control.

### 2.9. GP5+/GP6+ PCR

The primers used for HPV PCR were a single pair of consensus GP5+/GP6+ (150 bp), as previously described [4]. PCR was carried out in a reaction volume of 50 *μ*L containing 50 mM KCl, 10 mM Tris HCl (pH 8.3), 200 *μ*M each deoxynucleoside triphosphate, 3.5 mM MgCl_2_, 1 unit of thermostable DNA polymerase (Ampli*Taq*; Perkin-Elmer; Foster City, CA), and 50 pmol each of the GP5+ (5′-TTTGTTACTGTGGTAGATACTAC-3′) and GP6+ (3′-CTTATACTAAATGTCAAATAAAAAG-5′) primers. Samples were denatured for 4 minutes at 94°C and then underwent 40 cycles of amplification with a PCR processor (PE9600; Perkin-Elmer). Each cycle consisted of a denaturation step (1 minute at 94°C), a primer annealing step (2 minutes at 40°C), and a chain elongation step (1.5 minute at 72°C). Complete extension of amplified DNA was ensured by prolongation of the final elongation step by 4 minutes [29]. 

Negative and positive controls were included for each reaction.

PCR products were layered on 1.5% agarose gel and transferred onto positively charged nylon membranes (Qiabrane; Westburg) by diffusion blotting in 0.5 N NaOH-0.6 M NaCl.

DNA purification by gel extraction was done for samples that tested weakly positive.

A BLAST search was performed to assign sequences to known HPV types.

### 2.10. Gel Extraction Protocol

Gel extraction was performed with the QIAquick Gel Extraction Kit (Qiagen Inc., Valencia, CA), according to the supplied protocol. Briefly, DNA fragments were excised from the agarose gel with a clean, sharp scalpel. Gel slices were weighed in a colorless tube, and 3 volumes of buffer QG were added to 1 volume of gel. Gel was then dissolved via incubation at 50°C for 10 minutes. DNA was bound to the supplied column by centrifugation for 1 minute followed by discarding of flow-through. 0.5 of buffer QG was added to each sample, and flow-through discarded once more after centrifugation for 1 minute. 0.75 mL of buffer PE was added to the column and flow-through discarded after centrifugation for further washing. Columns were centrifuged again at 17,900 ×g and DNA was eluded by addition of 50 *μ*L of elution buffer (10 mM tris-Cl, pH 8.5) to the central membrane of the column followed by centrifugation for 1 minute.

### 2.11. Statistical Analysis

Locoregional control (LRC) was defined as time elapsed between initial diagnosis and development of recurrent locoregional disease. Overall survival (OS) was defined as time elapsed between diagnosis and death from any cause, and disease-free survival was defined as time from initial diagnosis to tumour recurrence.

Statistical analysis was performed using Fisher's test for categorical data and Kaplan-Meier's curves and log-rank statistics for disease-free survival, overall survival and locoregional control according to HPV status (and HPV genotype), treatment period, chemotherapy regimen, TNM stage, tumour site, and patient age. Multivariate analysis using COX models was used to adjust for imbalances in the aforementioned prognostic factors between groups. Fisher's exact test was also performed to determine the difference in acute toxicities (cutaneous toxicity, mucitis, nausea, and vomiting, as well as grade 3-4 neutropenia) according to HPV status. 

Smoking status was excluded from our analyses because the data were inconsistently recorded in our database.

This protocol was approved by our institution's ethics committee.

## 3. Results

### 3.1. Patient Characteristics

Prospective data on efficacy and toxicity was available for 560 patients treated with concomitant CRT. All patients had histological confirmation of SCC based on histological features in hematoxilin and eosin-stained tissue sections diagnosed by a pathologist experienced in head and neck pathology. From these 560 patients, 270 fixed and paraffin-embedded specimens were collected. Sufficient tissue for DNA extraction was present in 255 samples.

Of these 255 patients, 79.61%  (*n* = 203) were male and 20.39%  (*n* = 52) female. Patient characteristics are listed in Table 1. Two hundred and ten (82.25%) initially presented with stage IV cancers. Primary tumour sites are listed in Table 1; the oropharynx was the most common primary site, comprising 66.27% of cases. HPV prevalence was 68.6% and 53.3% for HPV 16 specifically in our patient population.

Median followup was 4.69 years.

### 3.2. HPV Testing

Prior to HPV DNA detection, 26 samples underwent PCR for detection of *β*-globin using the PCO_4_/GH_2_O method (also included in the LA-HPV Detection kit) to ensure that DNA suitable for detection with the LA-HPV method was present, and to control for the absence of competing inhibitors. 24 samples tested positive for *β*-globin. 

Combining the results of HPV DNA detection with the LA-HPV and GP5+/GP6+ detection methods, 175 samples (68.63%) tested positive for HPV DNA. HPV 16 was identified in 138 samples (78.86%).

255 samples were tested for the presence of HPV DNA using the LA-HPV detection method. Of these, 127 samples tested positive for the presence of HPV DNA. HPV 16 was the only genotype in 109 samples; coinfection was found in 6 samples. Among theses, coinfection with HPV 16 and HPV 18 was present in 3 cases; the others were co-infected with HPV 16 and HPV 84 (*n* = 1), HPV 16 and HPV 11 (*n* = 1), and HPV 33 and HPV 35 (*n* = 1). The other genotypes detected were HPV 18 (*n* = 4), HPV 33 (*n* = 3), HPV 35 (*n* = 3), HPV 26 (*n* = 1), and HPV 58 (*n* = 1).

The 128 samples that tested negative for HPV DNA with the LA method were tested for the presence of HPV DNA using the GP5+/GP6+ detection method, using a set of shorter primers (150 bp), to ensure that negative results were not caused by excessive DNA fragmentation due to prolonged preservation in paraffin. 48 samples tested positive for the presence of HPV DNA. DNA was present in sufficient amounts to be submitted for sequencing in 24 samples, for which a BLAST search was performed to assign sequences to known HPV types. HPV 16 was identified as the single genotype present in all 24 samples.

Among the 128 samples that tested negative for HPV DNA using the LA-HPV detection method, 27 were judged invalid as *β*-globin was not detected by the test, suggesting that the samples did not contain DNA suitable for analysis. Of these 27 samples, 16 also tested negative for HPV DNA using the GP5+/GP6+ detection method; these samples were tested for the presence of *β*-globin using PCO_3_/PCO_4_ probes (110 bp). All 16 tested positive for *β*-globin, confirming that negative results were unlikely to result from excessive DNA fragmentation. 

### 3.3. Survival According to TNM Stage

**Figure 1:**
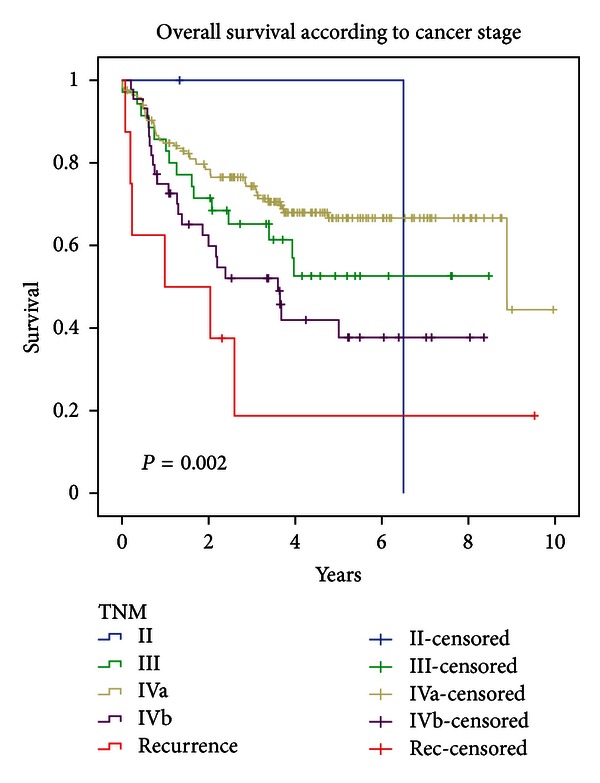
Overall survival according to TNM.

Overall survival was statistically significantly different based on TNM (log-rank *P* = 0.0017; cf.  Figure 1); OS was also statistically significant according to T (log-rank *P* < 0.0001) and N (log-rank *P* = 0.0112) separately.

DFS and LRC were also significantly different according to TNM (*P* = 0.0046 and *P* = 0.0150, resp.).

### 3.4. Survival According to HPV by Primary Subsite

**Figure 2:**
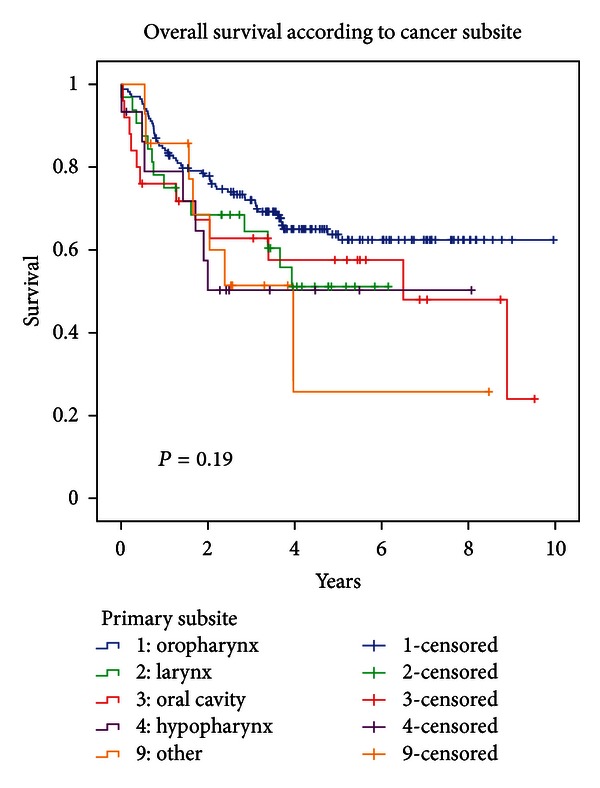
Overall survival according to primary subsite.

**Figure 3:**
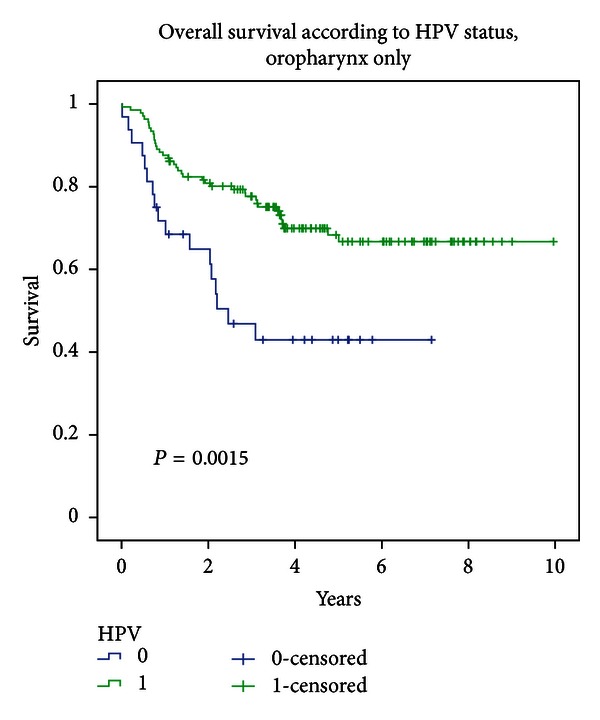
Overall survival for oropharyngeal primaries according to HPV status.

Overall survival was not statistically significantly different according to primary site (*P* = 0.187; cf.  Figure 2), nor was LRC (*P* = 0.0651). This is probably related to the fact that many cancer subsites were rare in our population. We therefore decided not to conduct a statistical analysis of interaction using the terms “primary site ∗ HPV status.” However, within the largest subgroup of patients suffering from oropharyngeal HNSCC, survival was significantly different according to HPV positivity (*P* = 0.002). Patients with HPV− oropharyngeal SCC had a median overall survival of 2.46 years, while median survival was not reached at a minimum of 4.63 years of followup for HPV-positive patients (cf.  Figure 3).

### 3.5. Efficacy Parameters According to HPV Status

**Figure 4:**
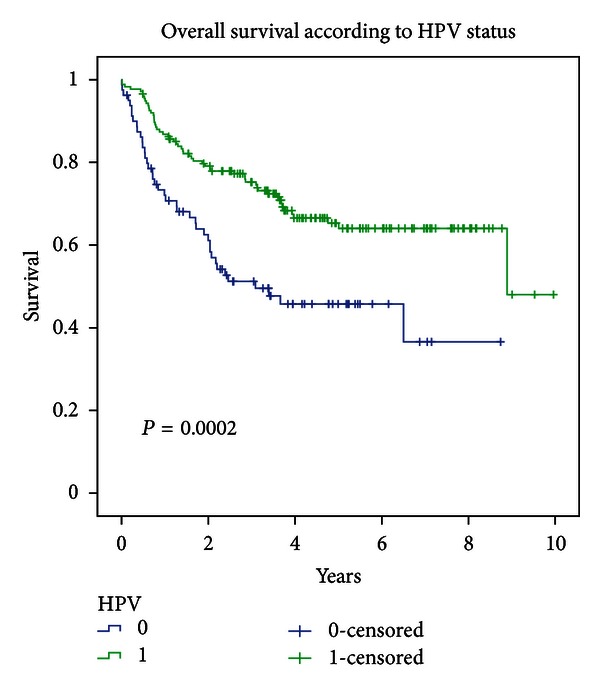
Overall survival according to HPV status.

For HPV+ and HPV− cases, respectively, median overall survival was 8.89 and 3.09 years (*P* = 0.0002) (cf.  Figure 4). This trend was also observed, and statistically significant, for HPV 16+ versus HPV 16− cases (log-rank *P* = 0.0005). Since there were statistically significant differences between the HPV+ and HPV− populations, a COX analysis adjusting for age, T, N, and treatment period (i.e., before and after 2001) and regimen was conducted and showed that the difference in overall survival remained significant (HR = 0.45; 95% CI = [0.289, 0.701]; *P* = 0.0004).

Disease-free survival (DFS) for HPV+ and HPV− cases was 8.89 years and 2.10 years, respectively (log-rank *P* = 0.0014). For HPV 16+ cases specifically and HPV 16− cases, respectively, median DFS was 8.89 and 3.53 years (log-rank *P* = 0.0010).

As for OS, the difference in DFS remained significant after adjustment for T, N, age, and treatment period and regimen (HR = 0.52; 95%  CI = [0.333, 0.818]; *P* = 0.0048).

LRC for HPV-negative and HPV-positive cases was 2.17 years and 8.89 years, respectively (*P* = 0.0002). For HPV 16-negative cases, median LRC was 3.09 years, while it was not reached for HPV 16-positive cases (*P* = 0.0001). This persisted on multivariate analysis (HR = 0.44; 95%  CI = [0.289, 0.679]; *P* = 0.0002).

There was no statistically significant difference in acute treatment-related toxicity between the two groups in the thirty days following treatment (data not shown). Toxic effects that were evaluated included cutaneous toxicities, mucitis, nausea and vomiting, need for gavage, grade 3-4 neutropenia, per-treatment hospitalisation, and per-treatment deaths. 

### 3.6. Survival Based on HPV Genotype

**Figure 5:**
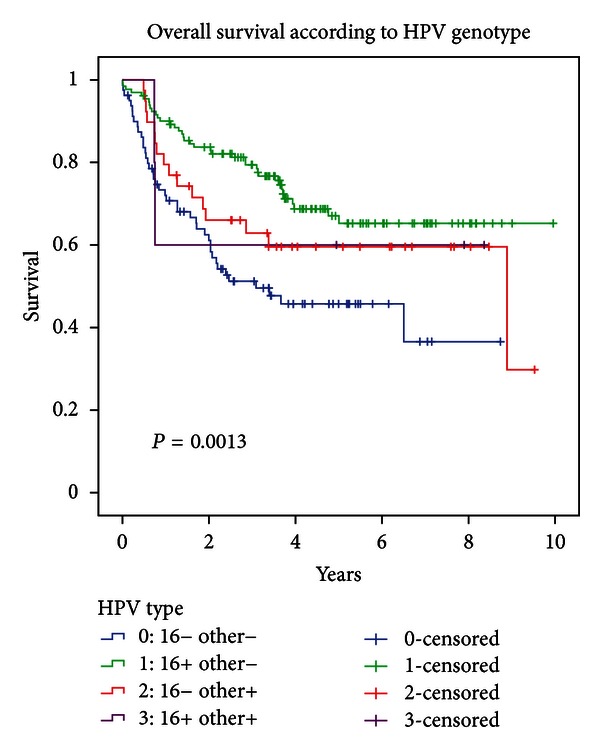
Overall survival according to HPV genotype.

Overall survival, DFS, and LRC were statistically significantly different based on HPV genotype (log-rank *P* = 0.0013, *P* = 0.0061, and *P* = 0.0008, resp.; cf.  Figure 5) but this was essentially driven by the large differences between the HPV16 positive group compared to the HPV-negative group, with the other subgroups being small.

### 3.7. Survival According to Chemotherapy Regimen

**Figure 6:**
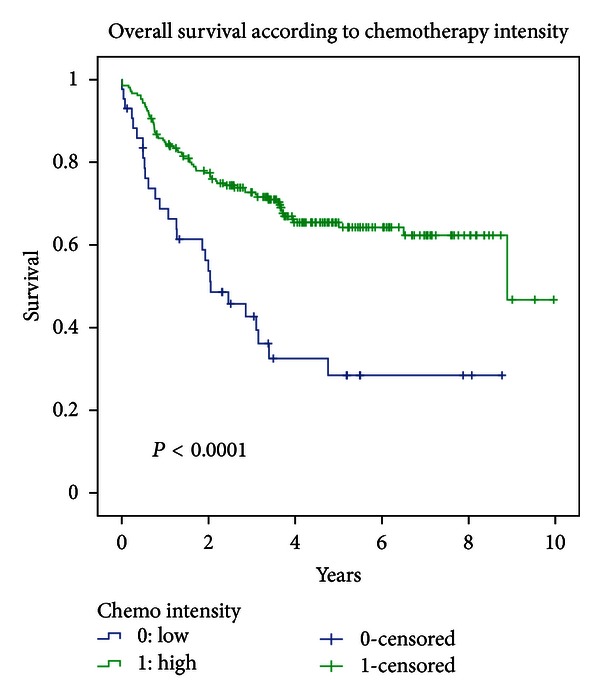
Overall survival according to chemotherapy regimen.

Overall survival was statistically significantly different based on chemotherapy regimen (*P* < 0.0001; cf.  Figure 6).

For patients receiving high-dose chemotherapy, that is, concurrent chemotherapy (Cisplatin 100 mg/m^2^ q 3 weeks × 3 or Carboplatin 70 mg/m^2^ d1-4 + 5-FU 600 mg/m^2^ d1-4 q 3 weeks × 3) median overall survival was 8.89 years; while median OS was 2.04 years for patient receiving low-dose (Cisplatin 6 mg/m^2^ daily or Carboplatin 25 mg/m^2^ daily), chemotherapy. 

DFS was also 8.89 years for patients receiving high-dose chemotherapy and 1.63 years for patients receiving low-dose chemotherapy (*P* = 0.0001).

Median LRC was 8.89 years and 1.92 years for patients receiving high- and low-dose chemotherapy, respectively (*P* < 0.0001).

HPV subgroup remains a statistically significant predictor of survival even in the high-dose chemotherapy group (which includes most of the cohort) with a median OS of 6.5 years in the high-dose chemotherapy HPV-negative group compared to a median OS not reached in the high-dose chemotherapy HPV-positive subgroup (*P* = 0.0006). This effect was present although not statistically significant in the low-dose chemotherapy, probably due to the small number of patients in that subgroup (*n* = 43).

Treatment regimen was modified from low-dose chemotherapy to high-dose chemotherapy in 2001; our results also show that OS, DFS, and LRC are statistically significantly different according to treatment period, that is, before and after 2001 (data not shown).

## 4. Discussion

Previous studies have reported improved outcomes in HPV-associated oropharyngeal cancer [5, 18, 30, 31], but need to be interpreted with caution as samples were often small, comprised patients who were not treated in a uniform fashion, and data were often collected retrospectively. Our data confirm the improved outcomes in terms of OS, DFS, and LRC for patients with HPV-positive HNSCC observed in retrospective studies. Moreover, our analysis confirms the prognostic impact of HPV positivity because treatment was similar for all patients in our cohort. HPV can thus be seen as strong positive prognostic factor even though no specific mechanism has been identified to explain higher rates of response to chemoradiation in spite of genetically distinct characteristics [32]. HPV-positive HNSCC patients seem to experience greater local-regional control; this could be due to a higher intrinsic sensitivity to radiation or better radiosensitisation with cisplatin. However, HPV-positive HNSCCs demonstrate a favourable prognosis regardless of treatment modality (surgery, radiation therapy, concurrent chemoradiation as in this study, or induction chemotherapy plus concurrent chemoradiation therapy) [5, 6, 22–24, 33]. This is consistent with the theory that HPV-positive and -negative tumours are different biological entities [34]. 

HPV prevalence may have been underestimated in our trial because of the reduced sensitivity of PCR in FFPE samples [35]. However, previous studies have demonstrated that a higher sensitivity was achieved by using PCR for HPV detections than that with other methods such as ISH or p16 immunohistochemistry [25, 36], though the gold standard for defining a tumour as being associated with HPV remains the detection of expression of HPV oncogenes E6 and E7 [32, 37].

Other studies have suggested that the biological behaviour of HPV-positive tumours may be altered by tobacco use [3, 4], possibly because these tumours are at higher risk for both local recurrence and distant metastases [38]. Indeed, genetic mutations induced by tobacco-related carcinogens may render HPV-positive tumours less responsive to therapy [39]. Previous studies have also demonstrated that tobacco smoking was associated with overall survival, and progression-free survival, with risks of death and cancer recurrence increasing for each additional pack-year of tobacco smoking; this effect has been shown to be similar for patients with HPV-positive and HPV-negative cancers [26]. These data were however excluded from our study as smoking status was inconsistently recorded in our database.

An increasing proportion of oropharyngeal cancer is linked to HPV, which, along with tobacco use, is the strongest independent determinant of OS in OSCC patients treated with chemoradiotherapy [26, 39]. Though our results are consistent with an increased response to chemoradiation therapy in HPV-positive HNSCC, our data neither confirm nor infirm that HPV-related HNSCC can be treated with a less stringent therapy and do not necessarily represent evidence for a difference in natural history between HPV-negative and HPV-positive cancers in the absence of treatment. A combination of tumour HPV status, pack-years of tobacco smoking, and cancer stage could be used to classify patients' risk of death [26]. 

In this cohort, treatment with 3-week high-dose chemotherapy proved to be more advantageous. This regimen is the most widely used chemotherapy regimen in combination with radiotherapy [40, 41]. Previous studies on daily low-doses (i.e., conventional doses) have led to mitigated outcomes, with results less conclusive than those using the cisplatin 100 mg/m^2^ regimen (for a cumulative dose of 300 mg/m^2^) [41, 42]. Treatment regimen was modified from low-dose chemotherapy to high-dose chemotherapy in our centre in 2001; our results also show that OS, DFS, and LRC are statistically significantly different according to treatment period, that is, before and after 2001 (data not shown).

This large study, with a cohort from one centre, confirms that HPV status is strongly associated with improved prognosis among H&N cancer patients receiving CRT and should be a stratification factor for all clinical trials including HNSCC cases. Separate trials in HPV-positive and HPV-negative oropharyngeal cancers will be needed to device the optimal treatment for each of these distinct entities, with the focus in HPV-positive cancers being to determine whether a decrease in treatment intensity and consequential toxicity can be achieved without compromising currently achieved outcomes. The comparison for a new therapy could consist of a concomitant boost-accelerated-fractionation regimen of radiotherapy or a standard-fractionation regimen, combined with concurrent, high-dose cisplatin, as both methods lead to similar results in terms of overall survival [26]. 

## Figures and Tables

**Table 1:** Patient characteristics according to HPV status.

Patient characteristic	Number of patients			
	HPV+	HPV−	*P *	Total
Number of patients	175	80		
Age (years)				
Median	55.42	59.56	*t*-test *P* = 0.0086	57.00
Range	25.25–75.03	39.64–78.72		25.25–78.72
T (*n* = 255)				
T1	36 (20.57%)	7 (8.75%)	Fisher *P* = 0.0203	43 (16.86%)
T2	39 (22.29%)	17 (21.25%)		56 (21.96%)
T3	48 (27.43%)	26 (32.50%)		74 (29.02%)
T4	51 (29.14%)	25 (31.25%)		76 (29.80%)
TX	0	1 (1.25%)		1 (0.39%)
Recurrence	1 (0.57%)	4 (5.00%)		5 (1.96%)
N (*n* = 254)*				
N0	15 (8.62%)	11 (13.75%)	Fisher *P* > 0.1	26 (10.24%)
N1	25 (14.37%)	10 (12.50%)		35 (13.78%)
N2	0	1 (1.25%)		1 (0.39%)
N2a	27 (15.52%)	10 (12.50%)		37 (14.57%)
N2b	42 (24.14%)	16 (20.00%)		58 (22.83%)
N2c	36 (20.69%)	22 (27.50%)		58 (22.83%)
N3	29 (16.67%)	10 (12.50%)		39 (15.35%)
TNM stage (*n* = 255)				
I	0	0	Fisher *P* > 0.1	0 (0%)
II	0	2 (2.5%)		2 (0.78%)
III	24 (13.71%)	11 (13.75%)		35 (13. 73%)
IVa	117 (68.86%)	49 (61.25%)		166 (65.10%)
IVb	30 (17.14%)	14 (17.50%)		44 (17.25%)
Recurrence	4 (2.29%)	4 (5.00%)		8 (3.14%)
KPS (*n* = 212)*				
60	1 (0.67%)	0	Fisher *P* = 0.1	1 (0.47%)
70	3 (2.00%)	1 (1.61%)		4 (1.89%)
80	30 (20.00%)	11 (17.74%)		41 (19.34%)
90	99 (66.00%)	49 (79.03%)		148 (69.81%)
100	17 (11.33%)	1 (1.61%)		18 (8.49%)
Chemotherapy (*n* = 254)*				
Daily Carboplatin or Cisplatin	17 (9.71%)	10 (12.66%)	Fisher *P* = 0.00061	27 (10.63%)
Daily Carboplatin + 5FU	113 (64.57%)	33 (41.77%)		146 (57.48%)
Cisplatin q 1 week or q 3 weeks	45 (25.71%)	36 (45.57%)		81 (31.89%)
Radiotherapy (*n* = 254)*				
Conventional	143 (82.18%)	77 (96.25%)	Fisher *P* = 0.0017	220 (86.28%)
IMRT	31 (17.82%)	3 (3.75%)		34 (13.33%)
Primary (*n* = 255)				
Oropharynx	137 (78.29%)	32 (40.00%)	Fisher *P* < 0.0001	169 (66.27%)
Larynx	14 (8.00%)	18 (22.50%)		32 (12.54%)
Oral cavity	9 (5.14%)	16 (20.00%)		25 (9.80%)
Hypopharynx	6 (3.43%)	9 (11.25%)		15 (5.88%)
Nasopharynx	6 (3.43%)	2 (2.50%)		8 (3.14%)
Paranasal sinuses	2 (1.14%)	1 (1.25%)		3 (1.18%)
Nose	1 (0.57%)	1 (1.25%)		2 (0.78%)
Unknown	0	1 (1.25%)		1 (0.39%)

*Indicates missing data.
